# APPLYING TIME-OF-FLIGHT SENSOR IN EARLY MOBILIZATION INTERVENTION FOR HOSPITALIZED OLDER ADULTS

**DOI:** 10.1093/geroni/igae098.2445

**Published:** 2024-12-31

**Authors:** I-Ling Chen, Po-Lung Yang, Hsiu-Yun Lai, Mu-Cyun Wang, Chien-Jung Pien, Han-Yueh Kuo

**Affiliations:** University of Washington, Seattle, Washington, United States; National Taiwan University Hospital, Taipei, Taipei, Taiwan (Republic of China); National Taiwan University Hospital, Hsin-Chu Branch, HsinChu, Hsinchu, Taiwan (Republic of China); National Taiwan University Hospital, Taipei, Taipei, Taiwan (Republic of China); National Taiwan University Hospital, Hsin-Chu Branch, HsinChu, Hsinchu, Taiwan (Republic of China); National Taiwan University Hospital, Hsin-Chu Branch, HsinChu, Hsinchu, Taiwan (Republic of China)

## Abstract

Early mobilization is crucial in preventing functional decline associated with hospitalization among older adults. To assess the effectiveness of an early mobilization intervention program, this randomized trial implements Time-of-Flight (ToF) sensor technology in quantifying patient mobility. Twenty-five adults aged 65 or above who were hospitalized for acute illness were enrolled in the study. The intervention group received early rehabilitation treatment by physical therapists, while the control group received education on self-exercises. All enrolled patients were monitored with Time-of-Flight sensors to record the number of out-of-bed movements during their hospital stay. Patient mobility scores were calculated upon enrollment and the date of discharge using the de Morton Mobility Index(DEMMI). The patients were followed up by phone call at 1 month and 6 months after discharge, collecting information about re-admission to the hospital and mortality. Results showed that both intervention and control groups improved upon discharge. The average increase of DEMMI score is higher in the intervention group(mean: 9.82; SD: 10.97) than in the control group (mean: 7.07; SD: 10.72). However, no statistical significance was detected(p=0.53). DEMMI scores positively correlated with average daily out-of-bed movements (Pearson correlation coefficient > 0.7 and p < 0.001), validating the measurements of ToF sensors. Our study has strengths in utilizing sensor technology that does not require wearable devices to obtain objective measurements of mobility. However, this study is limited by its small sample size. Future research is needed to further examine the effectiveness of early mobilization among hospitalized older adults in the clinical settings.

